# Genome-Wide Identification of the WD40 Gene Family in Tomato (*Solanum lycopersicum* L.)

**DOI:** 10.3390/genes14061273

**Published:** 2023-06-15

**Authors:** Cunyao Yan, Tao Yang, Baike Wang, Haitao Yang, Juan Wang, Qinghui Yu

**Affiliations:** 1 Institute of Horticulture Crops, Xinjiang Academy of Agricultural Sciences (Key Laboratory of Genome Research and Genetic Improvement of Xinjiang Characteristic Fruits and Vegetables), Urumqi 830000, China; yancunyao111@163.com (C.Y.);; 2 The State Key Laboratory of Genetic Improvement and Germplasm Innovation of Crop Resistance in Arid Desert Regions (Preparation), Urumqi 830000, China; 3 College of Horticulture, Xinjiang Agricultural University, Urumqi 830000, China

**Keywords:** WD40 gene family, tomato, phylogenetic, fruit development, bioinformatics keyword

## Abstract

WD40 proteins are a superfamily of regulatory proteins widely found in eukaryotes that play an important role in regulating plant growth and development. However, the systematic identification and characterization of WD40 proteins in tomato (*Solanum lycopersicum* L.) have not been reported. In the present study, we identified 207 *WD40* genes in the tomatoes genome and analyzed their chromosomal location, gene structure and evolutionary relationships. A total of 207 tomato *WD40* genes were classified by structural domain and phylogenetic tree analyses into five clusters and 12 subfamilies and were found to be unevenly distributed across the 12 tomato chromosomes. We identified six tandem duplication gene pairs and 24 segmental duplication pairs in the *WD40* gene family, with segmental duplication being the major mode of expansion in tomatoes. Ka/Ks analysis revealed that paralogs and orthologs of WD40 family genes underwent mainly purifying selection during the evolutionary process. RNA-seq data from different tissues and developmental periods of tomato fruit development showed tissue-specific expression of *WD40* genes. In addition, we constructed four coexpression networks according to the transcriptome and metabolome data for WD40 proteins involved in fruit development that may be related to total soluble solid formation. The results provide a comprehensive overview of the tomato WD40 gene family and will provide valuable information for the validation of the function of tomato *WD40* genes in fruit development.

## 1. Introduction

WD40 protein (also known as WD repeat protein) is a superfamily of regulatory proteins widely found in eukaryotes [1]. The protein consists of multiple WD repeat motifs containing 44–60 amino acid residues [2]. The WD repeat motifs have a glycine histidine dipeptide (Gly-His, GH) at the N-terminal and a tryptophan aspartate dipeptide (Trp-Asp and WD) at the C-terminal [3]. Each WD repeat motif forms a four-stranded antiparallel β-sheet structure in the folding of the protein, allowing the amino acid N and C termini to form a strong hydrogen bonding network to maintain the stability of the WD40 protein repeat fold [4]. Usually, WD40 structural domains consisting of four to eight WD repeats form β-propeller structures, while seven-bladed β-propeller proteins consisting of seven WD repeats (or multiples of seven) dominate among the identified WD40 structures and identified WD40 proteins [5].

WD40 family genes have a low level of sequence conservation and a high degree of cellular functional diversity. Some *WD40* genes have additional structural domains such as the LisH, TATA-box, COP1, FBOX, and CAF1 domains. Based on these structural domains, WD40 family genes can be divided into 32 functional subfamilies [6] which are involved in signal transduction [7,8], histone modification [9], cell cycle regulation [10,11], nuclear fusion [12], RNA processing [13], apoptosis [14], cytoskeleton assembly [15,16], transcriptional regulation [17], and other functions. WD40 proteins also play an important role in plant growth and development [18], fruit development [19], and hormone response through protein‒protein interactions as a scaffold for complex assembly [20]. For example, WD40 (TTG1) interacts with bHLH and MYB transcription factors to form the MBW (MYB-bHLH-WD40) complex in the regulation of fruit anthocyanin biosynthesis, trichome formation, and seed dormancy [21,22,23]. GTS1 (WD40) coordinates with Nop16 and L19e to regulate seed germination and biomass accumulation [24]. DWA1 (WD40) interacts with ABT (WD40) and interacts with ABI2 to regulate plant tolerance in response to salt stress and drought stress [25].

Tomato is a vegetable crop that is widely grown worldwide and occupies an important position in global agricultural production [26]. With the rise in living standards, the demand for tomato quality is increasing, and it is important to improve the quality of tomato fruit by understanding and analyzing the underlying mechanisms of tomato fruit development [27]. The *WD40* gene plays an important role in a variety of physiological processes in plants. In tomatoes, although the potential role of the *SlWD40* (*Solyc04g005020*) gene in tomato development has been intensively studied, a systematic study of the tomato WD40 gene family has not been reported [28]. In this study, we comprehensively analyzed the physicochemical properties, gene structure, motif composition, chromosomal location, gene duplication and evolutionary relationships of 207 *WD40* genes in tomatoes. The expression pattern of *WD40* genes during tomato fruit development was also analysed. In addition, WGCNA association analysis was performed to reveal the genetic network associated with the total soluble solid formation in tomato *WD40*-specific gene members during fruit development. The results of this study can provide valuable information for screening tomato WD40 genes that affect fruit quality during fruit development.

## 2. Materials and Methods

### 2.1. Identification of the Tomato WD40 Family

To identify the tomato *WD40* genes, the tomato ITAG 4.0 protein sequence was downloaded from the tomato genomics database Sol Genomics Network (https://solgenomics.net/projects/tomatodisease/, accessed on 18 December 2022) [29]. The Hidden Markov Model (HMM) for the WD40 protein (PF00400) was downloaded from the Pfam database, and the candidate WD40 protein sequence was obtained by comparing the tomato protein sequence by HMMER (v3.3.2) software [30]. The default parameter cut-off was set to 0.01. The candidate sequence was validated by the InterPro (https://www.ebi.ac.uk/interpro/, accessed on 18 December 2022) and SMART (http://smart.embl-heidelberg.de/smart/batch.pl, accessed on 18 December 2022) databases as the tomato WD40 protein sequence [31,32]. In addition, we downloaded the protein sequences of eggplant (*Solanum melongena*), pepper (*Capsicum annuum*) and tobacco (*Nicotiana benthamiana*) from Sol Genomics Network (https://solgenomics.net/projects/tomatodisease/, accessed on 25 May 2023), using the *Arabidopsis* WD40 protein sequence as a query sequence, a BLASTP search (e-value: 1 × 10^−5^) was performed and the WD40 members of Solanaceae were later obtained by HMMER validation [33,34]. Information on the number of amino acids, molecular weight and isoelectric point of the tomato WD40 protein sequence was analyzed using Expasy (https://web.expasy.org/protparam/, accessed on 20 December 2022). The subcellular localization of tomato WD40 protein was predicted using WoLF PSORT (https://wolfpsort.hgc.jp/, accessed on 22 December 2022) [35].

### 2.2. Classification and Gene Structure Analysis of WD40 Gene

According to the results of InterPro identification of the structural domain of the WD40 protein, subfamily classification of WD40 was performed. The conserved motifs in the tomato WD40 protein sequence were identified using MEME (https://meme-suite.org/meme/tools/meme, accessed on 25 December 2022) and the number of motifs was set to 10 [36]. The exon‒intron structure of the tomato *WD40* genes was visualized with TBtools [37]. The promoter sequence (start codon) of the *WD40* genes in tomato was extracted 1500 bp upstream, and the cis-regulatory elements (CARE) of the *WD40* genes were predicted by the Search for CARE tool in the PlantCARE database (https://bioinformatics.psb.ugent.be/webtools/plantcare/html/, accessed on 27 December 2022) [38].

### 2.3. Chromosomal Localization and Gene Duplication in Tomato

The distribution of the tomato *WD40* genes across the 12 tomato chromosomes was analyzed based on the positional annotation information of the *WD40* genes obtained from the annotation file Gff of the ITAG 4.0 tomato genome. The genome and annotation files of *Arabidopsis* TAIR10 version were downloaded from the TAIR database (https://www.arabidopsis.org/index.jsp, accessed on 27 December 2022). The potato PGSC v4.03 genome and annotation files were downloaded from the Spud DB database (http://spuddb.uga.edu/, accessed on 27 December 2022). Eggplant (*S. melongena* V4.1), pepper (*C. annuum* ‘Dempsey’ v1.0) and tobacco (*N. benthamiana* v2.6.1) genomes and their annotation files were downloaded from Sol Genomics Network (https://solgenomics.net/projects/tomatodisease/, accessed on 27 May 2023). Intraspecific replication events and interspecific collinear analysis of the tomato WD40 genes were performed using MCScanX software with e-value set to 1 × 10^−10^ [39]. Where the *Arabidopsis* and potato WD40 genes used for interspecies analysis were from identified WD40 genes [38,40]. The gene duplication events were visualized using CIRCOS and TBtools software [37,41]. To further assess the selection pressure on WD40 collinear gene pairs, we extracted cds sequences and protein sequences of WD40 genes within and between species and aligned them by MUSCLE. Ka (nonsynonymous)/Ks (synonymous) was then calculated using ParaAT 2.0. KaKs_Calculator has been embedded in ParaAT 2.0 [42,43]. The command we executed in this study is “ParaAT.pl -h Solanaceae collinearity.tab -n Solanaceae.cds.fa -a Solanaceae.pep.fa -p proc -m muscle -f axt -g -k -o result_dir”.

### 2.4. Phylogenetic Analysis of the Tomato WD40 Gene

Multiple sequence alignment of tomato and *Arabidopsis* WD40 protein sequences was performed using MUSCLE, and the results were imported into MEGA 11 software to construct an unrooted evolutionary tree of the WD40 family [44]. The algorithm used was the neighbor-joining (NJ) model, the validated bootstrap value was set to 1000, and the model selection parameter was Poisson.

### 2.5. Mining and Identification of SSRs in Tomato WD40 Genes

We used the Web Simple Sequence Repeats (SSR) Finder tool in the PSSRD database (http://www.pssrd.info/, accessed on 10 January 2023) to identify SSR sequences with 1, 2, 3, 4, 5, 6, 7, 8, 9 and 10 nucleotide repeats in the tomato WD40 gene (referring to the parameters described by Gao et al. [45,46].

### 2.6. Identification of miRNAs Targeting WD40 Genes in Tomato

The miRNAs that might target the tomato *WD40* genes were predicted using psRNATarget (https://www.zhaolab.org/psRNATarget/home, accessed on 10 January 2023) (using the default parameters) [47], and the interaction network was visualized using Cytoscape tools (version 3.8.0) [48].

### 2.7. Tomato RNA-Seq Data Analysis

We obtained tomato RNA-seq data from previous research to study the expression pattern of the tomato WD40 gene in different tissues and fruit development stages (Table S1) [49]. These data were calculated using transcripts per kilobase million (TPM) method. Tissue samples of root, stem, leaf, flower and fruit (10 d, 20 d, 30 d, 35 d, 40 d, 43 d, 47 d, 50 d and 55 d after flowering) were collected from the tomato cultivar ‘MicroTom’ at day 45 after sowing, with three independent biological replicates at each stage [49].

### 2.8. Weighted Gene Coexpression Network Analysis (WGCNA)

Metabolomic data for nine ‘MicroTom’ tomato fruit developmental stages (including data related to sucrose, glucose, glucoronic acid, gluconic acid, citramalate, malic acid, citric acid, trigalacturonic acid, inositol and lycoperodine) were obtained from previous studies [49] (Table S2). WGCNA association analysis was performed using the R program package WGCNA shiny (https://github.com/ShawnWx2019/WGCNA-shinyApp, accessed on 15 January 2023) to analyze TPM values and metabolites from nine tomato fruit developmental periods to assess the possible involvement of *WD40* genes in the regulatory network related to the formation of total soluble solids during fruit development. The TPM expression matrix was normalized by logTPM (Data Filter: Remove genes that have a logTPM of less than 1 in more than 90% of the samples). The power of β = 12 (scale-free R^2^ = 0.79) was chosen as a soft threshold to ensure a scale-free network. The minimum module size was 30, allowing the merging of modules. We visualized the network connections using Cytoscape tools (version 3.8.0) [48].

### 2.9. Plant Material and Expression Analysis

The plant material used in this study was the tomato cultivar *S. lycopersicum* ‘MicroTom’. ‘MicroTom’ plants were grown in a growth chamber at 23 ± 2 °C under a light/dark photoperiod of 16/8 h. Tomato fruits were harvested at d 10, 20, 30, 40 and 50 after flowering (a flowering rate of more than 50% was considered the blooming period, 45 DPG), and the harvested fruits were frozen in liquid nitrogen and then transferred to −80 °C. At each time point, at least three different biological samples were collected for subsequent experiments. Total RNA was extracted using a polysaccharide polyphenol kit (TIANGEN, Beijing, China). qPCR and One-Step RT MasterMixes (Abm, Richmond, BC, Canada) were used to synthesize first-strand cDNA. Gene-specific primers designed with Primer3Plus (http://www.bioinformatics.nl/cgi-bin/primer3plus/primer3plus.cgi/, accessed on 10 January 2023) (Hung and Weng, 2016) (Table S3) were used. Quantitative PCR (qPCR) was performed using SYBR qPCR Master Mix (Vazyme, Nanjing, China). The *SlActin* gene was used as an internal reference. Each treatment contained three technical replicates and each replicate included at least three fruits. Ct values of the WD40 gene were assessed using the $2^{-∆∆Ct}$ method [50].

## 3. Results

### 3.1. Identification of Tomato WD40 Protein

To exclude sequences that did not contain typical *WD40* structural domains, we conducted a search using HMMER software and performed the validation using InterPro (pfam00400) and SMART (SM000320). Ultimately, 207 tomato WD40 genes were obtained. In addition, we retrieved 920 WD40 protein sequences from three solanaceae species (pepper, tobacco and eggplant) (Table S4).

**Figure 1:**
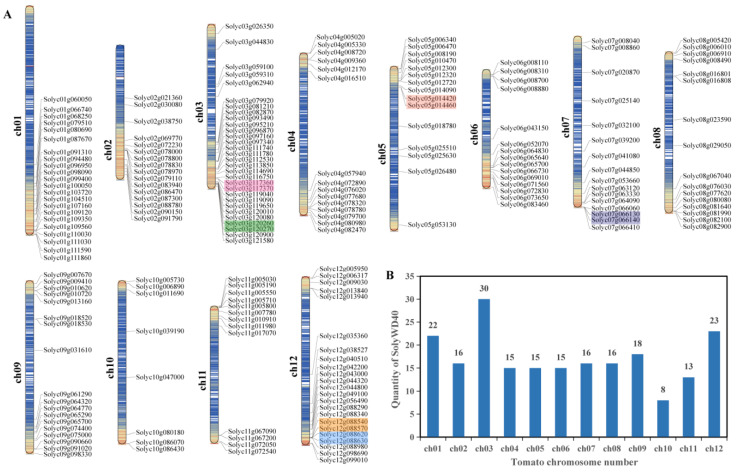
Chromosomal distribution of WD40 family genes in tomato. (**A**) Distribution of tomato WD40 genes on 12 chromosomes. Tandemly duplicated genes are marked with colored boxes. (**B**) Number of tomato WD40 genes on each chromosome. Blue lines in chromosome segments represent low gene density and red lines represent high gene density.

The tomato WD40 protein had an aliphatic index range of 52.85 (Solyc08g067040) to 117.25 (Solyc09g018520) and a GRAVY value range of −0.805 (Solyc08g067040) to 0.738 (Solyc09g018520) (Table S5). The subcellular localization results showed that 111 tomato WD40 proteins were located in the nucleus, 44 tomato WD40 proteins were located in the cytosol, 31 tomato WD40 proteins were located in the chloroplast, 8 tomato WD40 proteins were located in the mitochondria, 5 tomato WD40 proteins were located in the cytoskeleton, 4 tomato WD40 proteins were located in the plasma membrane, 2 tomato WD40 proteins were located in the vacuolar membrane, 1 tomato WD40 protein was located in the endoplasmic reticulum and 1 tomato WD40 protein was located in the cell wall (Table S5). A chromosomal localization analysis showed that the 207 WD40 genes were widely and unevenly distributed across the 12 chromosomes (Figure 1A), with the highest number on chromosome 3 and the lowest number on chromosome 10 (Figure 1B).

### 3.2. Classification and Structural Analysis of the WD40 Protein Subfamily

**Figure 2:**
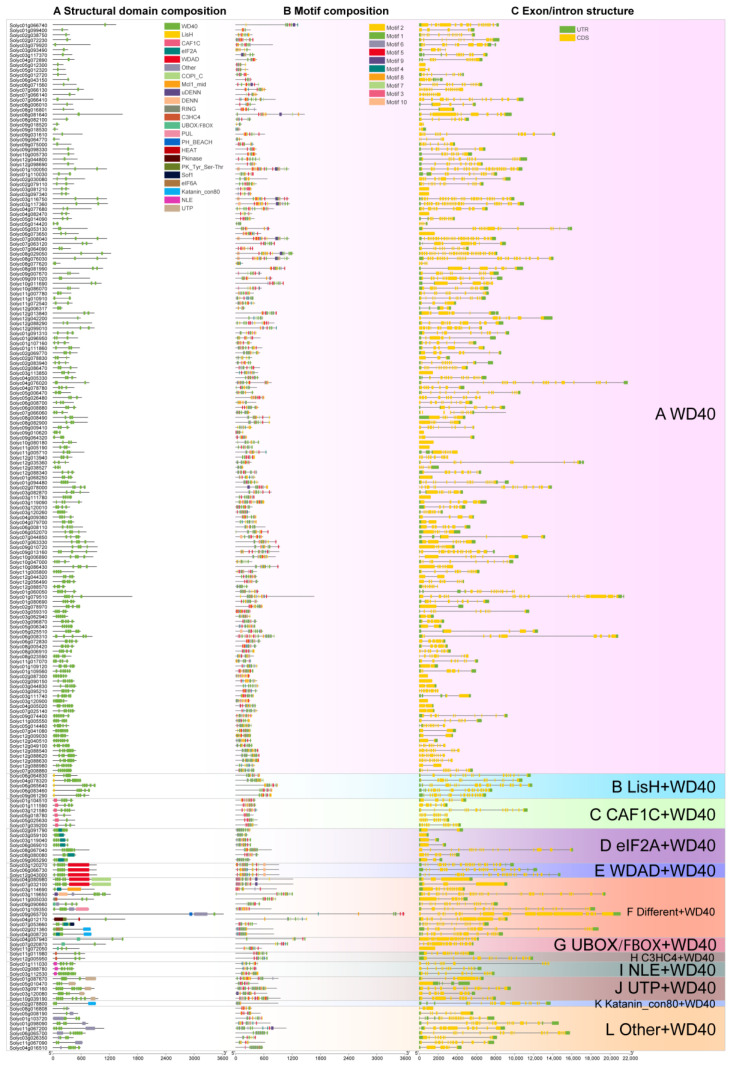
Gene structure and conserved motif analysis of tomato WD40 proteins. (**A**) Structural domain type and distribution in tomato WD40 protein. (**B**) Distribution of conserved motifs in tomato WD40 protein. (**C**) Exon/intron structure of the tomato WD40 genes.

The WD40 structural domains in the 207 tomato WD40 proteins identified ranged from one to eight, with some of the WD40 proteins containing other structural domains. Based on the types of these structural domains, we classified the 207 WD40 protein sequences into 12 subfamilies (Figure 2A). Of these, 151 of the WD40 proteins containing only the WD40 structural domain were classified into subfamily A. Five WD40 proteins containing the WD40 structural domain and the LisH structural domain were classified into subfamily B. Six *WD40s* containing only the WD40 structural domain and the CAF1C structural domain were assigned to subfamily C. Seven *WD40s* containing only the WD40 structural domain and the eIF2A structural domain were grouped into subfamily D. Three *WD40s* containing only the WD40 structural domain and the WDAD structural domain were assigned to subfamily E. Twelve *WD40s* containing multiple structural domains and WD40 structural domains were classified into subfamily F. Three *WD40s* containing only the WD40 structural domain and the UBOX/FBOX structural domain were classified into the G subfamily. Two *WD40s* containing only the WD40 structural domain and C3HC4 structural domain were assigned to subfamily H. Three *WD40s* containing only the WD40 and NLE structural domains were assigned to subfamily I. Five *WD40s* containing only the WD40 and UTP structural domains were grouped into subfamily J. One *WD40* containing only the WD40 structural domain and the Katanin_con80 structural domain was classified into the K subfamily. Nine *WD40s* containing WD40 structural domains and other domains were classified into subfamily L.

We used the MEME online program to search for the conserved motifs shared by the *WD40* genes to further investigate their diversity in tomatoes. We identified 10 conserved motifs and named them Motif 1 to Motif 10 (Figure 2B, Table S6). Among the 10 motifs, Motif 1 was widely distributed among 204 tomato *WD40s* with 178 *WD40s* containing Motif 6, and with Motif 9 and Motif 10 being the least distributed with 15 *WD40s* containing Motif 9 and 11 *WD40s* containing Motif 10. In addition, we found that the *WD40* structural domains consisted of various combinations of motifs. Examples include Motif 1 and Motif 3, Motif 1 and Motif 6, Motif 2 and Motif 3, Motif 2 and Motif 8, Motif 3 and Motif 4, Motif 5 and Motif 8 and Motif 7 and Motif 8. Whether the order of these specific motif arrangements confers a unique functional role to the *WD40* gene requires further investigation (Figure S1).

To gain insight into the structure of tomato *WD40s*, we analyzed their exon and intron composition (Figure 2C) and found that the number of exons and introns varied greatly. The maximum number of exons in *Solyc08g029050* was 33 (32 introns), while 16 tomato *WD40s* had only one exon and no introns.

### 3.3. Analysis of Cis-Elements in the Promoters of WD40 Family Genes

**Figure 3:**
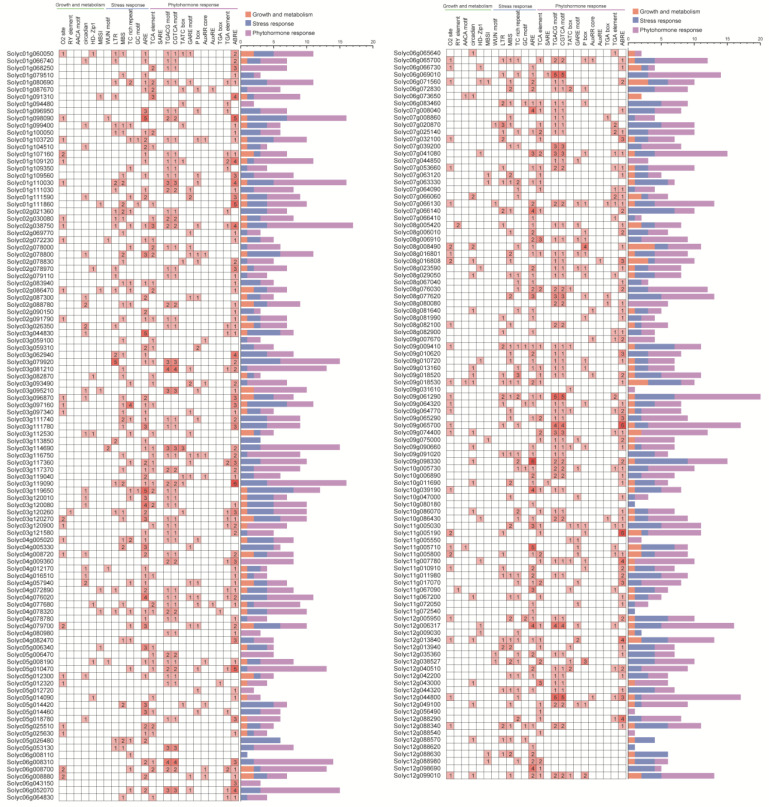
Cis-acting elements identified in the promoter of the tomato *WD40* gene family. The stacked graph on the right side represents the total number of promoter elements in each category.

To explore the mechanism of action of tomato WD40 family genes in stress response and development, cis-acting elements in the 1500 bp upstream of the *WD40* promoter were analyzed using the PlantCARE online tool (Figure 3). Six cis-acting elements were identified in the first category (plant growth metabolism), including the O2 site, involved in the regulation of maize alcohol-soluble protein metabolism; the RY element, involved in seed-specific regulation; the AACA motif, involved in endosperm-specific negative expression; the circadian, involved in circadian rhythm control; the HD Zip1, involved in fenestrated chloroplast differentiation; and the flavonoid biosynthesis MYB binding site I (MBSI). The largest proportion (42.3%) was accounted for by the O2 site.

In the second category (stress response), 6 cis-acting elements were identified, including the trauma response element WUN motif, the cis-element LTR involved in the low-temperature response, the cis-element MBS involved in drought induction, the cis element TC rich repeats involved in defense and stress response, the cis-element GC motif involved in hypoxia-specific induction and the cis-element ARE involved in anaerobic induction. The largest proportion (47.2%) was accounted for by ARE.

In the third category (phytohormone response), 12 cis-acting elements were identified, including the salicylic acid response element TCA element, the SARE, the MeJA response element TGACG motif, the CGTCA motif including the gibberellin response element TATC box, the GARE motif, the P box, the growth hormone response element AuxRR core, the AuxRE, the TGA-box, the TGA element, the abscisic acid response element ABRE and the largest proportion of ABRE (27.5%). The presence of these motifs indicates that *WD40* is widely involved in a variety of life activities such as growth and development and stress response in tomatoes.

### 3.4. Gene Duplication Analysis of WD40 Genes

**Figure 4:**
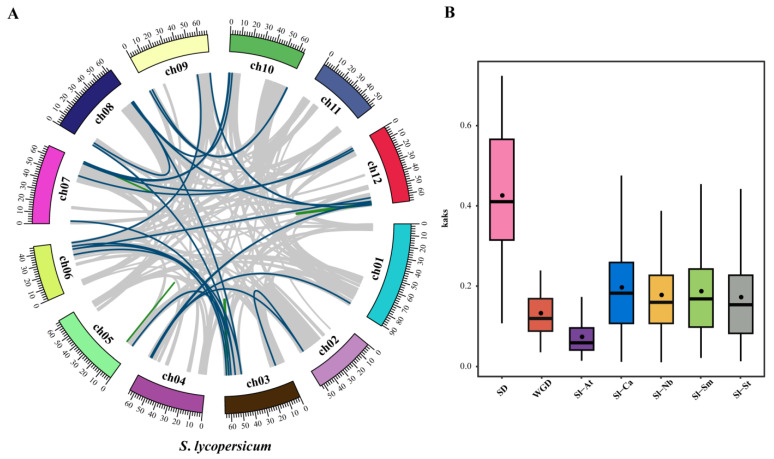
Covariance analysis and Ka/Ks ratio estimation of *WD40* family genes. (**A**) Collinear relationships of WD40 genes within tomato species. Blue lines indicate WD40 nodal repeat gene pairs and green lines represent WD40 tandem repeat gene pairs. (**B**) Box plot of nonsynonymous substitutions versus synonymous substitution ratios (Ka/Ks) in collinear *WD40* gene pairs.

Tandem and segmental duplications of tomato genes were identified using MCScanX software. A total of 30 duplication gene pairs (Figure 4A, Table S7) were identified in the tomato *WD40* genes including 6 tandem duplication gene pairs (20%) (Figure 1) and 24 segmental duplication pairs (80%) (Figure 4A). These results suggest that some tomato *WD40* genes may have arisen from gene duplication events and that segmental duplication events are the main driver of tomato *WD40* evolution.

To further explore the potential evolutionary process of the tomato *WD40* gene family, we constructed collinear relationships between tomato and *Arabidopsis* and between tomato and solanaceae species (pepper, potato, tobacco and eggplant). The results showed that there were 88 WD40 homologous gene pairs between tomato and *Arabidopsis*, 171 homologous gene pairs between tomato and pepper, 185 homologous gene pairs between tomato and potato, 300 homologous gene pairs between tomato and tobacco and 184 homologous gene pairs between tomato and eggplant. (Figure S2, Table S7). Interestingly, some collinear gene pairs of tomato *WD40* family genes only existed between tomato and *Arabidopsis* or between tomato and solanaceae species. For example, *Solyc03g095210* is collinear with *CaDEM03G21970*, *Niben261Chr06g0969002*, *PGSC0003DMT400013474* and *SMEL4.1_10g012510*, while *Solyc03g081210* is only collinear with *AT5G24520*. The formation of these specific collinear gene pairs may be related to an evolutionary mechanism of tomato plants. In the collinear analysis of *WD40* in tomato and solanaceae species (pepper, potato, tobacco and eggplant), *Solyc03g082870* was found to be collinear with 15 *WD40* genes of solanaceae species, indicating that *Solyc03g082870* may play an important role in *WD40* evolution.

The substitution rate (Ka/Ks) is an effective indicator to determine the selection pressure of repeated events, with Ka/Ks < 1 representing purifying selection, Ka/Ks = 1 representing neutral selection and Ka/Ks > 1 representing positive selection. Therefore, we calculated Ka/Ks for tomato *WD40* gene collinear gene pairs (Figure 4B). The Ka/Ks of tomato *WD40* tandem duplicated gene pairs ranged from 0.11 to 0.72 with a mean value of 0.43. The Ka/Ks of segmental duplicated gene pairs ranged from 0.04 to 0.31 with a mean value of 0.13. The Ka/Ks values of all tandem duplicated and segmental duplicated *WD40* gene pairs were less than one, which implies that these genes evolved under the influence of purifying selection. The average Ka/Ks value of tandem duplication genes (0.43) was higher than that of segmental duplication genes (0.13), indicating that tandem duplication evolved faster than other duplication events. The Ka/Ks values of tomato and *Arabidopsis* homologous gene pairs ranged from 0.01–0.24 with a mean value of 0.073. The Ka/Ks values of tomato and pepper homologous gene pairs ranged from 0.01 to 0.69 with a mean value of 0.191. The Ka/Ks values of tomato and potato homologous gene pairs ranged from 0.01–0.70 with a mean value of 0.172. The Ka/Ks values of tomato and tobacco homozygous gene pairs had Ka/Ks values ranging from 0.01 to 0.60 with a mean value of 0.177. Tomato and eggplant homozygous gene pairs had Ka/Ks values ranging from 0.02 to 0.60 with a mean value of 0.187. This suggests that the *WD40* genes have also been subject to purifying selection during the evolution of different species. 

### 3.5. Phylogenetic Analysis of the WD40 Genes

**Figure 5:**
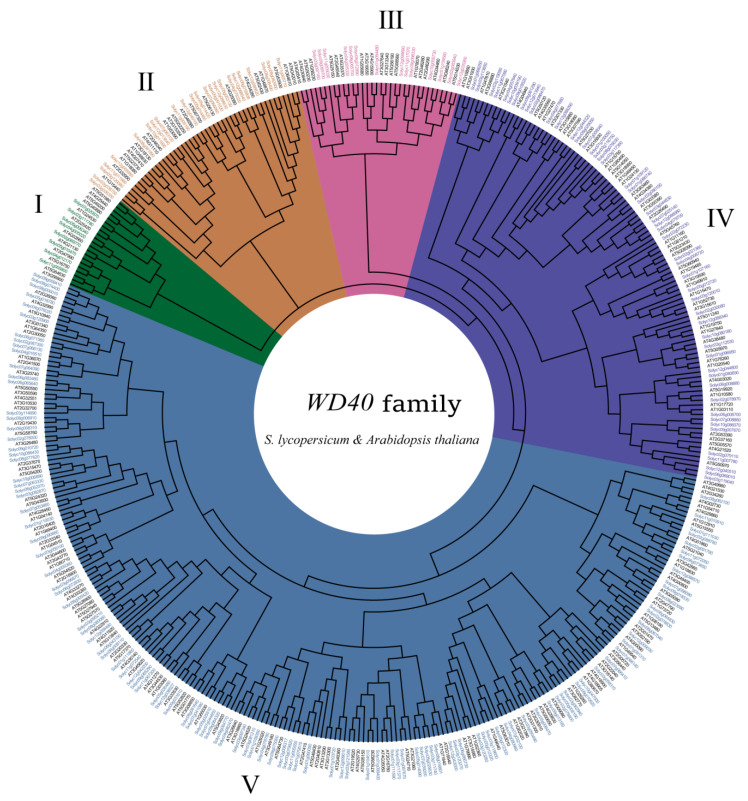
Phylogenetic relationships between tomato and *Arabidopsis* WD40 proteins. The Roman numerals represent the different clusters.

We constructed an unrooted phylogenetic tree containing 230 *Arabidopsis* WD40 proteins and 207 tomato WD40 proteins using the neighbor-joining (NJ) method (Figure 5), and we divided these WD40 proteins into five clades: Clade I contained 8 tomato and 12 *Arabidopsis* WD40 proteins, Clade II contained 24 tomato WD40 proteins and 22 *Arabidopsis* WD40 proteins, Clade III contained 14 tomato WD40 proteins and 20 *Arabidopsis* WD40 proteins, Clade IV contained 50 tomato WD40 proteins and 55 *Arabidopsis* WD40 proteins and Clade V contained 111 tomato WD40 proteins and 121 *Arabidopsis* WD40 proteins.

### 3.6. Identification of SSRs in Tomato WD40 Family Genes

A total of 35 SSR loci with sequence lengths between 20 bp and 58 bp were extracted from the tomato *WD40* genes (Table S8). These identified SSR loci were classified into mono-base repeat, di-base repeat, tri-base repeat, penta-base repeat, hexa-base repeat and hepta-base repeat sequences according to their types, with the largest proportion of SSR loci of double-base repeat type (16/35) and the smallest proportion of SSR loci of single-base repeat type (1/35).

### 3.7. Interaction of Tomato WD40 Genes with microRNA

To predict miRNAs that may target tomato *WD40*, we used the mRNA sequence of the tomato *WD40* gene as an input sequence to psRNATarget (Figure S3, Table S9). We identified a total of 110 miRNAs targeting the regulation of 174 *WD40* genes. The length of these miRNAs ranged from 20–24 nucleotides. The predicted targeting regulatory mechanism of miRNAs showed that a single miRNA could target and regulate multiple tomato *WD40* genes. For example, *sly-miR6022*, *sly-miR6024* and *sly-miR9476-3p* targeted and regulated 28 *WD40* genes, and *sly-miR482e-5p* targeted and regulated 25 *WD40* genes, while some miRNAs, such as *sly-miR166a*, *sly-miR166b*, *sly-miR166c-3p*, *sly-miR168a-5p*, *sly-miR168b-5p*, *sly-miR1916* and *sly-miR482b*, targeted and regulated only a single *WD40* gene. 

### 3.8. RNA-Seq Analysis

**Figure 6:**
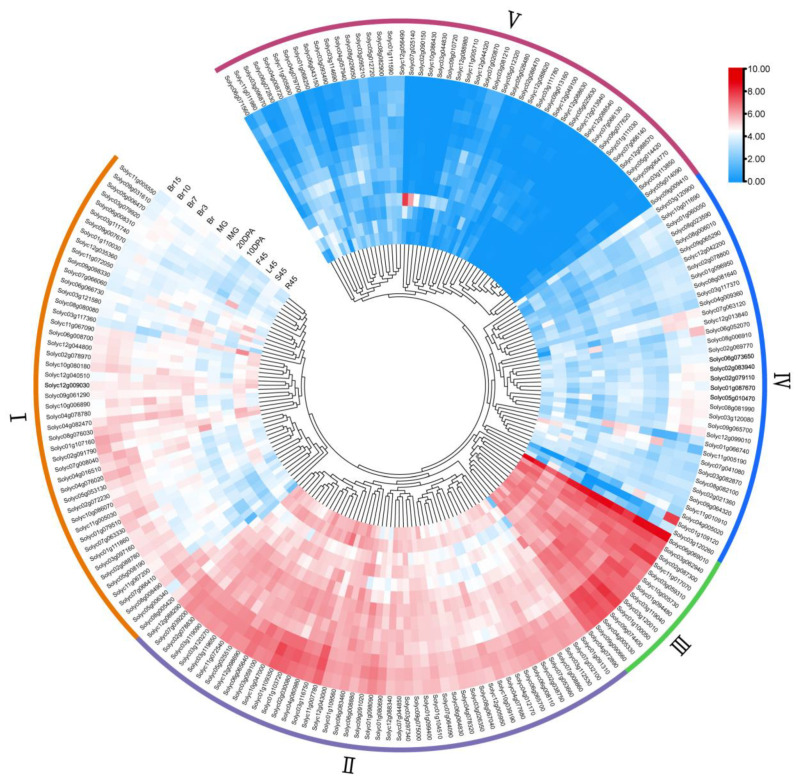
Expression of the tomato *WD40* gene family in different tissues. Heatmaps were created from publicly available transcriptome data for different tomato tissues. The color bars in the heatmap indicate the average of the TPM values of log-transformed *WD40* genes in the three biological replicate samples. R45, S45, L45 and F45 represent roots (R45), stems (S45), leaves (L45) and flowers (F45) collected on day 45. 10 DPA/20 DPA represents the developing fruit harvested 10/20 days after flowering. IMG and MG represent immature green fruits (30 d) and mature green fruits (35 d), respectively. Br, Br3, Br7, Br10 and Br15 represent the fruit collected at 0 d, 3 d, 7 d, 10 d and 15 d of the breaker period (40 d), respectively. Roman numerals represent different groupings.

To elucidate the tissue expression pattern of the tomato *WD40* genes, we downloaded RNA-seq data for ‘MicroTom’ tomato from a previous study and conducted an analysis of the spatial and temporal expression profiles of tomato *WD40s* in different tissues (including roots, stems, leaves and fruits) at different developmental stages (Table S1). The hierarchical clustering of gene expression profiles was generated by log2 normalization of the TPM values for 196 tomato *WD40s* (*Solyc02g078000*, *Solyc05g012300*, *Solyc05g014460*, *Solyc05g018780*, *Solyc08g016801*, *Solyc08g016808*, *Solyc09g010620*, *Solyc09g018520*, *Solyc09g018530*, *Solyc12g006317* and *Solyc12g038527* no transcripts detected). Based on the expression characteristics of tomato *WD40s* (Figure 6), the tissue expression profiles of tomato *WD40s* were clustered into 5 groups (groups I–V). Group I consisted of 48 genes whose expression levels ranged from 1.65 to 85.23 with a mean value of 20.62. Group II included 49 genes with high expression levels in most tissues, with expression levels ranging from 2.56 to 188.36 and a mean value of 45.58. Group III contained 13 genes with high expression in all 13 tissues with expression levels ranging from 37.62 to 440.18. Group IV had 37 genes with low expression levels in most tissues. Group V included 49 genes that were barely expressed in almost all the tissues tested except for individual tissues, and their mean value was 1.78.

### 3.9. WGCNA Analysis

**Figure 7:**
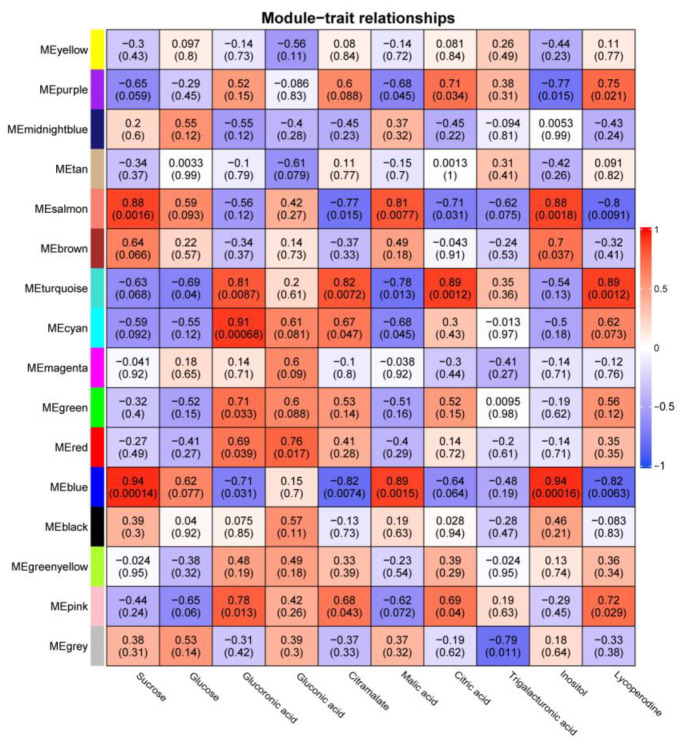
Correlation of coexpression modules with metabolites. The color of the heat map indicates the magnitude of the correlation. The numbers in the heat map are the correlation values, and the numbers in parentheses are the *p* values.

Coexpression networks are an effective way to identify gene clusters with similar functions, and we constructed coexpression networks for the transcriptome and metabolome of nine fruit developmental periods using the WGCNA shiny application. The transcriptome expression matrix was filtered through filters and the final 14,461 genes were assigned to 16 modules (Table S10), of which 147 tomato *WD40* genes were present in 11 modules (104 *WD40* genes in the MEturquoise module, 13 *WD40* genes in the MEblue module, 10 *WD40* genes in the MEbrown module, 5 *WD40* genes in the MEgreen module, 5 *WD40* genes in the MEyellow module, 3 *WD40* genes in the MEblack module, 2 *WD40* genes in the MEpink module, 2 *WD40* genes in the MEred module, 1 *WD40* gene in the MEcyan module, 1 *WD40* gene in the MEsalmon module and 1 *WD40* gene in the MEgreen–yellow module) (Figure 7).

We used the correlation coefficient |r| > 0.8 and significant *p* value < 0.01 as the basis for determining the correlation between modules and metabolites and found that four modules (MEturquoise, MEblue, MEcyan and MEsalmon) were highly correlated with seven metabolites (sucrose, glucoronic acid, citramalate, malic acid, citric acid, inositol and lycoperodine), with the MEturquoise module positively correlated with glucoronic acid, citramalate, citric acid and lycoperodine. The MEblue module was positively correlated with sucrose, malic acid and inositol and negatively correlated with citramalate and lycoperodine. The MEcyan module was positively correlated with glucoronic acid. The MEsalmon module was positively correlated with sucrose malic acid and negatively correlated with lycoperodine.

We visualized these four modules (Figures S4 and S5) using Cytoscape tools (version 3.8.0), where the MEturquoise module consisted of 6735 genes, the MEblue module consisted of 3151 genes, the MEcyan module consisted of 51 genes and the MEsalmon module consisted of 53 genes.

### 3.10. Analysis of the Tomato WD40 Gene Expression Patterns in Different Fruit Developmental Periods

**Figure 8:**
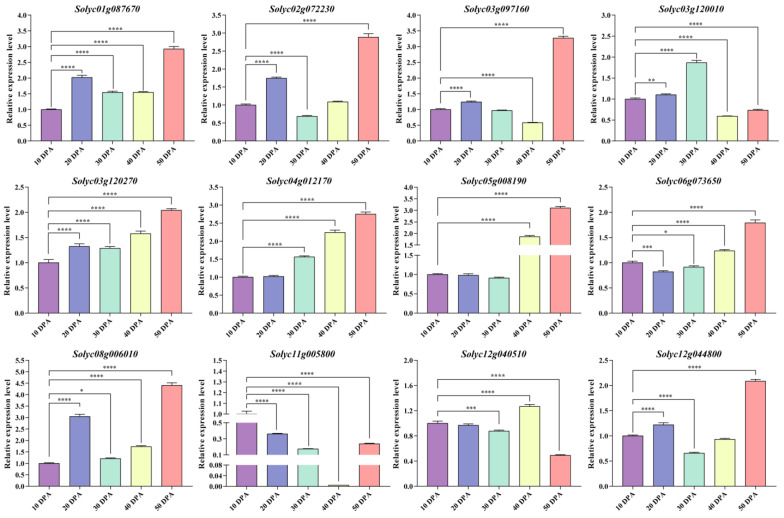
Expression analysis of 12 *WD40* genes in MicroTom tomato fruits. The horizontal coordinates of the graph are the five developmental periods of the fruit, and the vertical coordinates are the relative expression of each *WD40* gene. The error bars are the standard deviations of the three technical replicates. Statistically significant differences were determined according to one-way ANOVA (* *p* < 0.05, ** *p* < 0.01, *** *p* < 0.001, **** *p* < 0.0001).

We randomly selected 12 *WD40* genes from the WGCNA module for qRT-PCR experiments to assess changes in the *WD40* gene transcript levels during the developmental periods of ‘MicroTom’ tomato fruits (Figure 8). The results showed that all 12 selected *WD40* genes were significantly expressed during fruit development, with most *WD40* genes having the highest expression level at 50 days post-anthesis (DPA), In contrast, the expression level of the *Solyc03g120010* gene was highest at 30 DPA, while that of the *Solyc11g005800* gene was highest at 10 DPA and that of the *Solyc12g040510* gene was highest at 40 DPA. These results further indicate that the tomato *WD40* genes play an important role in fruit development.

## 4. Discussion

Tomato is an important cash crop. The *WD40* gene family has been reported to play an important role in a variety of physiological processes. However, there are relatively few studies on the whole genome of the *WD40* gene family in tomatoes. With the advent of the high-quality tomato genome SL4.0, a foundation has been laid for a comprehensive analysis of the *WD40* family of genes at the genome-wide level [51]. A total of 207 *WD40* members have been identified in tomato, which is more than the number of members in potato (178) [40] but fewer than the number of members in *Arabidopsis* (230) [33], mango (315) [52] and peach (220) [53], and the genome sizes of these five plants were found to be different: 758 Mb for tomato [51], 701 Mb for potato [54], 116 Mb for *Arabidopsis* [55], 489 Mb for mango [56] and 378 Mb for peach [57]. These findings indicate that there is no absolute correlation between the number of *WD40* superfamily members and the genome sizes of these species. Exon‒intron structural diversity is an important component of gene family evolution. In exon‒intron analysis, the number of introns is highly diverse (ranging from 0 to 32), and this diversity arises from exon/intron gain/loss, exonization/pseudoexonization and insertion/deletion during evolution. These three mechanisms give rise to divergence in gene structure and function [58].

To further elucidate the protein structure of tomato WD40s, we classified them based on the structural domain type. A total of 207 tomato WD40 proteins were broadly classified into 12 groups, with 72% of the WD40 proteins containing only the WD40 structural domain, accounting for the majority of all subgroups classified, a pattern that was also found in potato (72.4%) and rose (67.9%) [40,59]. Notably, even within the same subfamily, WD40 proteins are different in terms of sequence length, number of repeats and many other features. The reason for this may be due to the low sequence similarity between WDRs and the variable number of WDRs within a single WD40 structural domain. The conserved motifs of tomato WD40 proteins also differed greatly in number and order of arrangement. In terms of motif number, for example, Motif 1 was widely distributed among 204 tomato WD40 proteins, whereas Motif 10 was present in only 11 tomato WD40 proteins. In terms of motif order, the WD40 structural domain consisted of multiple motif combination, such as Motif 1 and Motif 3, Motif 1 and Motif 6, Motif 2 and Motif 3, Motif 2 and Motif 8, Motif 3 and Motif 4, Motif 5 and Motif 8 and Motif 7 and Motif 8. This result is consistent with the findings for the structural diversity of motifs in potatoes [40], and the difference in the number and order of these conserved motifs may be the main reason for the structural and functional diversity of WD40 proteins (Figure S1).

Cis-acting elements are important components of plant regulatory networks that help provide insight into transcriptional regulation and that reveal the function of related genes [60]. In this study, by predicting the promoter sequence of tomato *WD40* genes, 24 action elements mainly involved in growth metabolism, stress response and hormone response were identified, among which stress and hormone response elements were most widely distributed such as MBS, TGACG, CGTCA, GARE motif, ABREs, etc. The MBS element has been reported to be involved in the plant response to drought stress [61], the TGACG motif and CGTCA motif in the MeJA hormone response, the GARE motif in the gibberellin response and ABREs mainly in the ABA hormone response. These hormone-responsive processes can be indirectly involved in the plant response to abiotic and biotic stresses [62,63,64,65,66]. Thus, the analysis based on cis-acting elements suggests that these *WD40* genes are widely involved in the response of tomatoes to multiple stresses.

Tandem and segmental repeat patterns play an important role in the expansion of the tomato *WD40* family. In the present study, the number of tandem duplicated *WD40* genes accounted for 5.80% (12/207) of all *WD40* genes, while the number of segmental duplicated *WD40* genes accounted for 10.63% (22/207) of the total. A total of 88.24% (45/51) of the duplicated genes in the duplication events were from subfamily A based on structural domain classification. Therefore, gene duplication events appear to be the main driver for the evolution of tomato *WD40* subfamily A. This aligns with the results from a wheat study [67].

In general, immediate homologs clustered in a phylogenetic tree in a subgroup or subevolutionary branch have similar gene structures and functions. Therefore, we constructed an unrooted phylogenetic tree by the neighbor joining (NJ) method. The phylogenetic analysis classified all tomato WD40 proteins into five subgroups (clusters I to V), and this result was consistent with the WD40 classification of wheat, peach, and rose [53,59,67]. Each tomato WD40 protein could cluster with an *Arabidopsis* WD40 protein corresponding to the same evolutionary branch; therefore, these evolutionary branches could be used for a comparative analysis of tomato and *Arabidopsis* WD40 protein functions.

A large number of SSRs are distributed across eukaryotic genomes, and the use of SSR markers plays an important role in genetic diversity studies [68], comparative genomics [69], trait association analysis [70] and linkage mapping [71]. The development of SSR technology has facilitated the identification of functional genes in horticultural crops such as grapes [72], strawberries [73] and melons [74], which has accelerated the progress of genetic improvement and improved the selection and breeding of new cultivars. In this study, a total of 35 SSRs were identified from members of the tomato *WD40* gene family. The identification of these SSRs provides insights into additional possibilities of potential WD40 functions.

MicroRNAs (miRNAs) are a class of short, highly conserved endogenous noncoding small RNAs ranging from 19 to 24 nucleotides in length [75]. miRNAs function as a class of negative regulators that negatively regulate gene expression by targeting mRNA cleavage or translation inhibition, thereby playing a role in plant development and the response to environmental stress [76]. To elucidate the function and regulatory mechanisms of the tomato *WD40* gene, we predicted miRNAs that might target the tomato *WD40* gene. The prediction results showed 110 miRNAs targeting and regulating 174 *WD40* genes. These miRNAs have well-defined roles and functions in crop growth [77], fruit development [78], biological response [79], hormone synthesis [80] and stress response [81]. This miRNA prediction provides new insights for exploring WD40 functions. 

The analysis of published RNA-seq datasets from different tissues of tomato showed that many *WD40* genes were expressed in different tomato tissues, with some showing tissue specificity [49]. For example, the *Solyc06g069010* gene was highly expressed in all tissues, the *Solyc01g068250* gene was differentially expressed in roots, the *Solyc06g071560* gene was differentially expressed in stems and leaves, the *Solyc11g011980* gene was differentially expressed in leaves, the *Solyc07g025140* gene was differentially expressed in leaves and flowers, the *Solyc10g086430*, *Solyc02g090150*, *Solyc12g088980* genes were differentially expressed in flowers, the *Solyc04g079700* gene was differentially expressed in fruit development (20 d) and the *Solyc01g109120* gene was differentially expressed in the breaker stage (15 d). These differentially expressed genes suggest that different tomato *WD40* members perform different functions in different tissues and play important roles in the growth and development of tomatoes.

Fruit development is a process that is related to fruit quality. We used the WGCNA shiny application to correlate RNA-seq and metabolites in order to obtain the genetic network of tomato *WD40* genes associated with total soluble solids formation during fruit development. Among the selected metabolites, glucose and sucrose (soluble sugars), malic acid, citric acid, citramalate and trigalacturonic acid (organic acids) were the components of total soluble solids [82,83]. Glucoronic acid, gluconic acid inositol, and lycoperodine are closely related to the formation of total soluble solids [84,85]. In this study, four coexpression modules (MEturquoise, MEblue, MEcyan and MEsalmon) that were significantly associated with metabolites were obtained by expression matrix-trait association, where the MEturquoise module contained 104 *WD40* genes, the MEblue module contained 13 *WD40* genes, 1 *WD40* gene in the MEcyan module and 1 *WD40* gene in the MEsalmon module. We identified genes in the MEblue and MEturquoise modules that have been reported to be associated with total soluble solids in tomato. For example, the gene *PEPCK* (*Solyc04g076880*), involved in the sugar/acid ratio in ripe tomato fruit [86], *SPS* (*Solyc09g092130*, *Solyc11g045110*), involved in sucrose accumulation in tomato [87,88], and *AgpL1* (*Solyc01g109790*), which improves total soluble solids in fruit [89], were identified in the MEblue module. The gene *sucr* (*Solyc03g083910*), which controls sucrose accumulation [90], *GDH1* (*Solyc10g078550*), which regulates glucose and fructose content in tomato [91]; *bZIP1* (*Solyc08g005230*) and *bZIP2* (*Solyc08g082730*), which increase tomato fruit sugar content (sucrose/glucose/fructose) [92], and *invertase* (*Solyc04g081440*) and *sucrose synthase* (*Solyc02g081300*), which are genes involved in sucrose synthesis [93], were identified in the MEturquoise module. In addition, the cis-SV eQTL loci *Solyc01g101260* (*Solyc01G003449*) and *Solyc04g054930* (*Solyc04G001842*), which are associated with total soluble solids, were identified in the MEblue and MEturquoise modules, respectively [94]. These results suggest that tomato WD40 proteins may regulate metabolites through these coexpression networks and thus be involved in the formation of total soluble solids.

## 5. Conclusions

The present study provides the first comprehensive identification and analysis of the *WD40* gene family in tomatoes. A total of 207 *WD40* family members were identified in the tomato genome and were grouped into 5 clusters and 12 subfamilies. They were unevenly distributed across the 12 tomato chromosomes. Many plant growth metabolic elements, hormone response elements and stress response elements were identified in the promoter region. Moreover, segmental duplication was found to be the major mode of family expansion during the evolution of tomato *WD40*. Expression analysis indicated that *WD40* genes may be involved in the growth and development of tomato roots, stems, leaves, flowers and fruits. In addition, the coexpression network analysis constructed using WGCNA suggested that *WD40* may be involved in tomato fruit development with total soluble solid formation. These results lay the foundation for further studies on the functions of the *WD40* family in tomato fruit development and have application potential for tomato quality improvement breeding.

## Data Availability

The data presented in this study are available in the article and its Supplementary Materials.

## Supplementary Materials

The following supporting information can be downloaded at: https://www.mdpi.com/article/10.3390/genes14061273/s1, Figure S1: The motif of the tomato WD40 gene was linked to the expression matrix; Figure S2: Co-linear relationship of WD40 genes; Figure S3: Network diagram of miRNA-targeted regulation of the WD40 genes in tomato; Figure S4: Genes coexpressed with tomato WD40 gene in MEturquoise module and MEsalmon module; Figure S5: Genes coexpressed with tomato WD40 gene in MEblue module and MEcyan module. Table S1: TPM values of WD40 gene in RNA-seq data from different tissues of tomato; Table S2: Metabolite content of tomato at different fruit development periods; Table S3: List of primers used for qRT-PCR; Table S4: WD40 protein sequence of solanaceae species (pepper, tobacco, eggplant); Table S5: Basic information of WD40 genes identified in tomato; Table S6: Sequences of the 10 predicted motifs of the tomato WD40 protein; Table S7: Collinear relationships and KA/KS of WD40 genes in tomato, *Arabidopsis* and solanaceae species (pepper, potato, tobacco and eggplant); Table S8: SSR loci in the WD40 genes of tomato; Table S9: Predicting the interaction between tomato WD40 gene and microRNA; Table S10: Statistics of genes in the module.
