# Supplementary figures and images for: Genome-Wide Identification of the WD40 Gene Family in Tomato (Solanum lycopersicum L.)
Source: Genes (Basel). 2023 Jun 15;14(6):1273. doi: 10.3390/genes14061273 (PMC10298117; doi:10.3390/genes14061273)

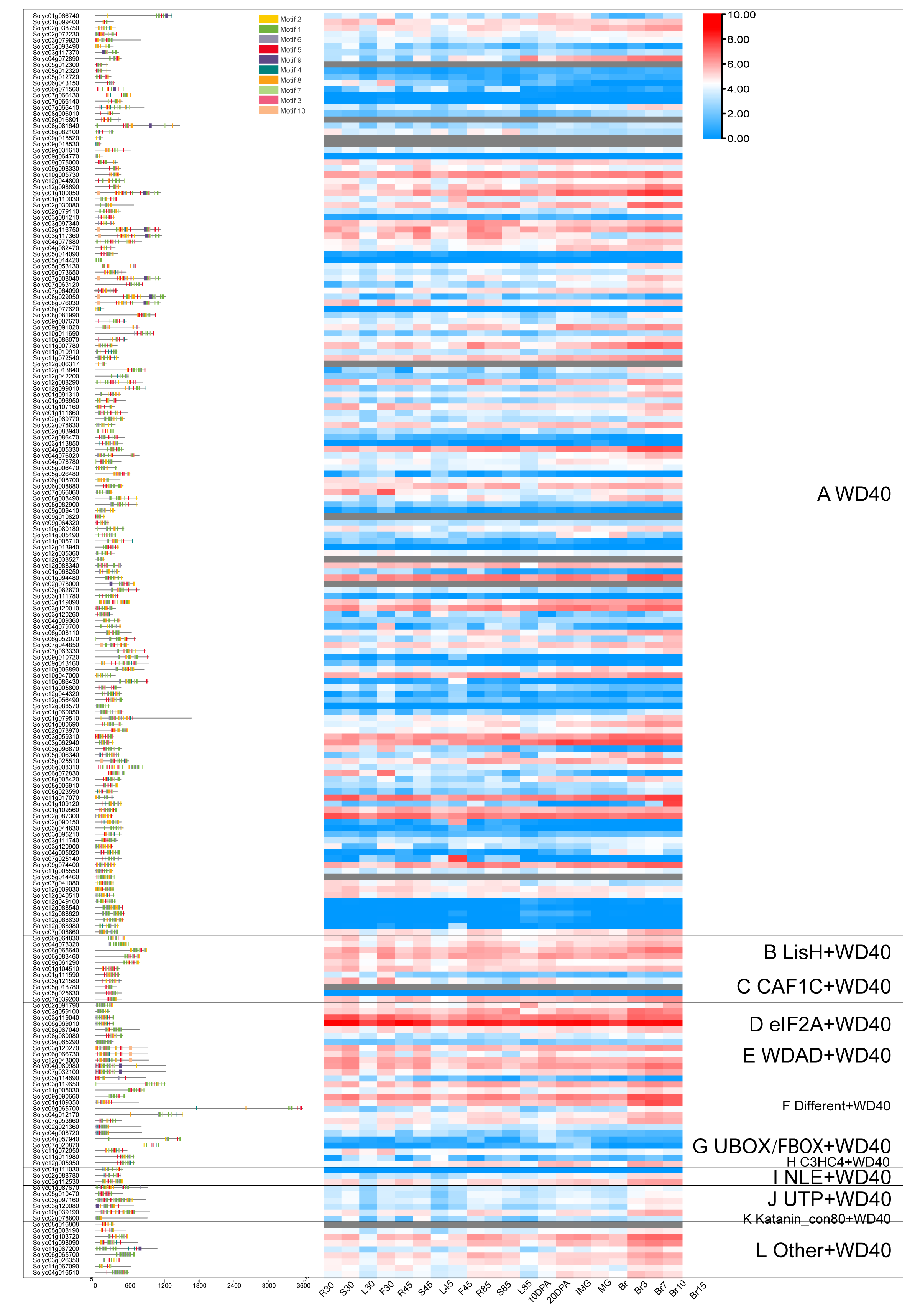

Supplement: Supplementary file 1 [file genes-14-01273-s001.zip › FigureS1 The motif of the tomato WD40 gene was linked to the expression matrix.tif]

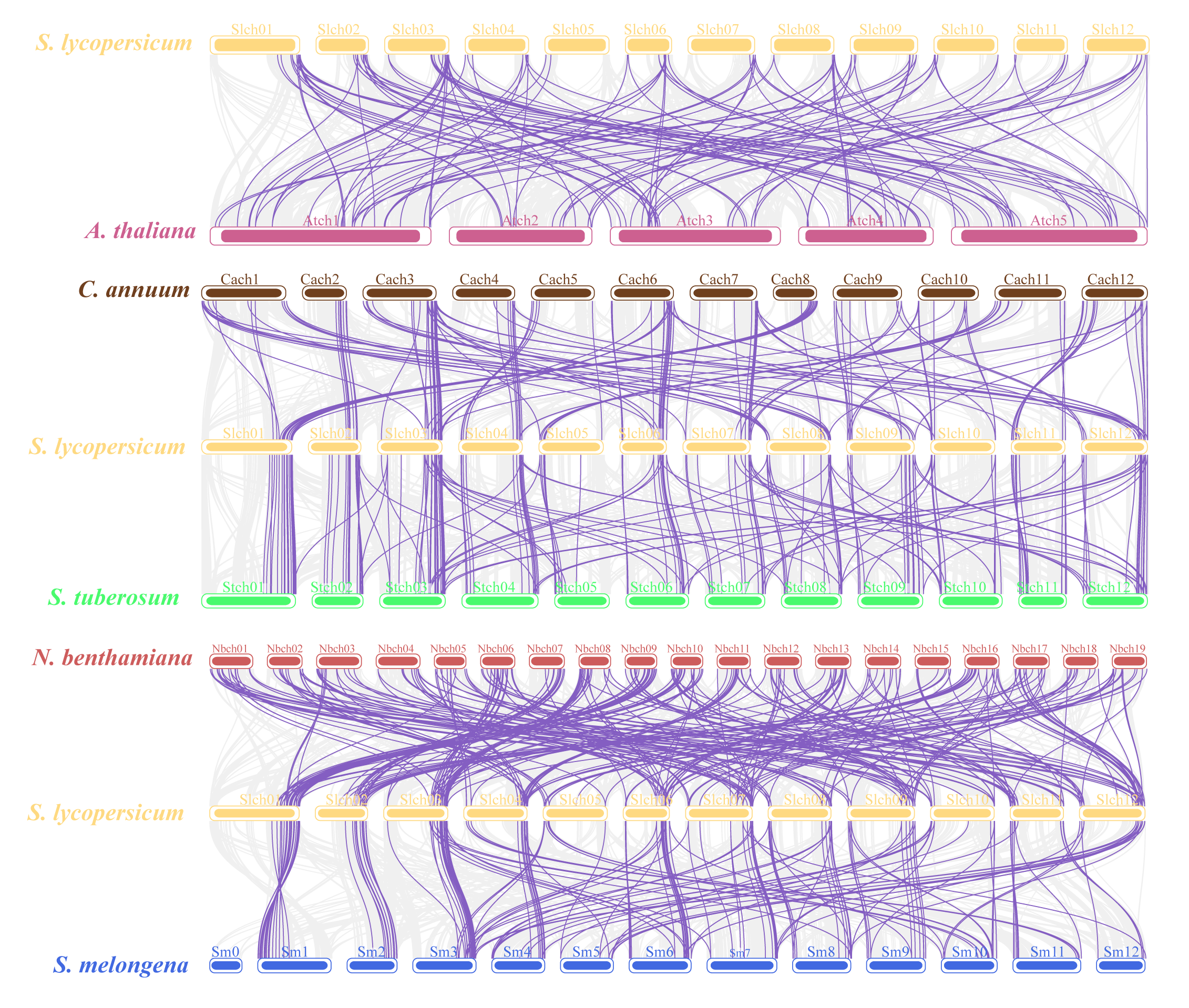

Supplement: Supplementary file 1 [file genes-14-01273-s001.zip › FigureS2 Co-linear relationship of WD40 genes.tif]

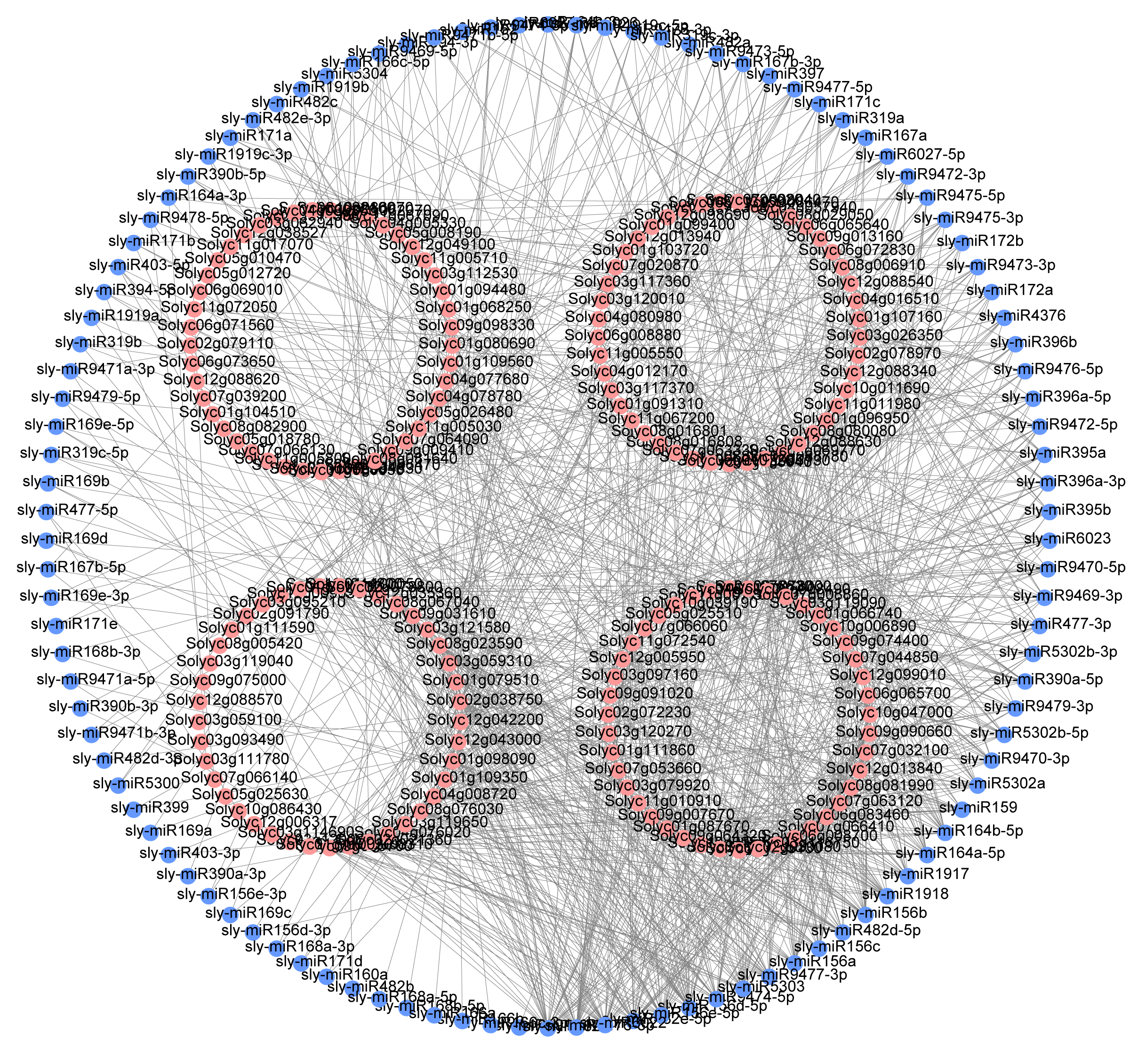

Supplement: Supplementary file 1 [file genes-14-01273-s001.zip › FigureS3 Network diagram of miRNA-targeted regulation of the WD40 genes in tomato.tif]

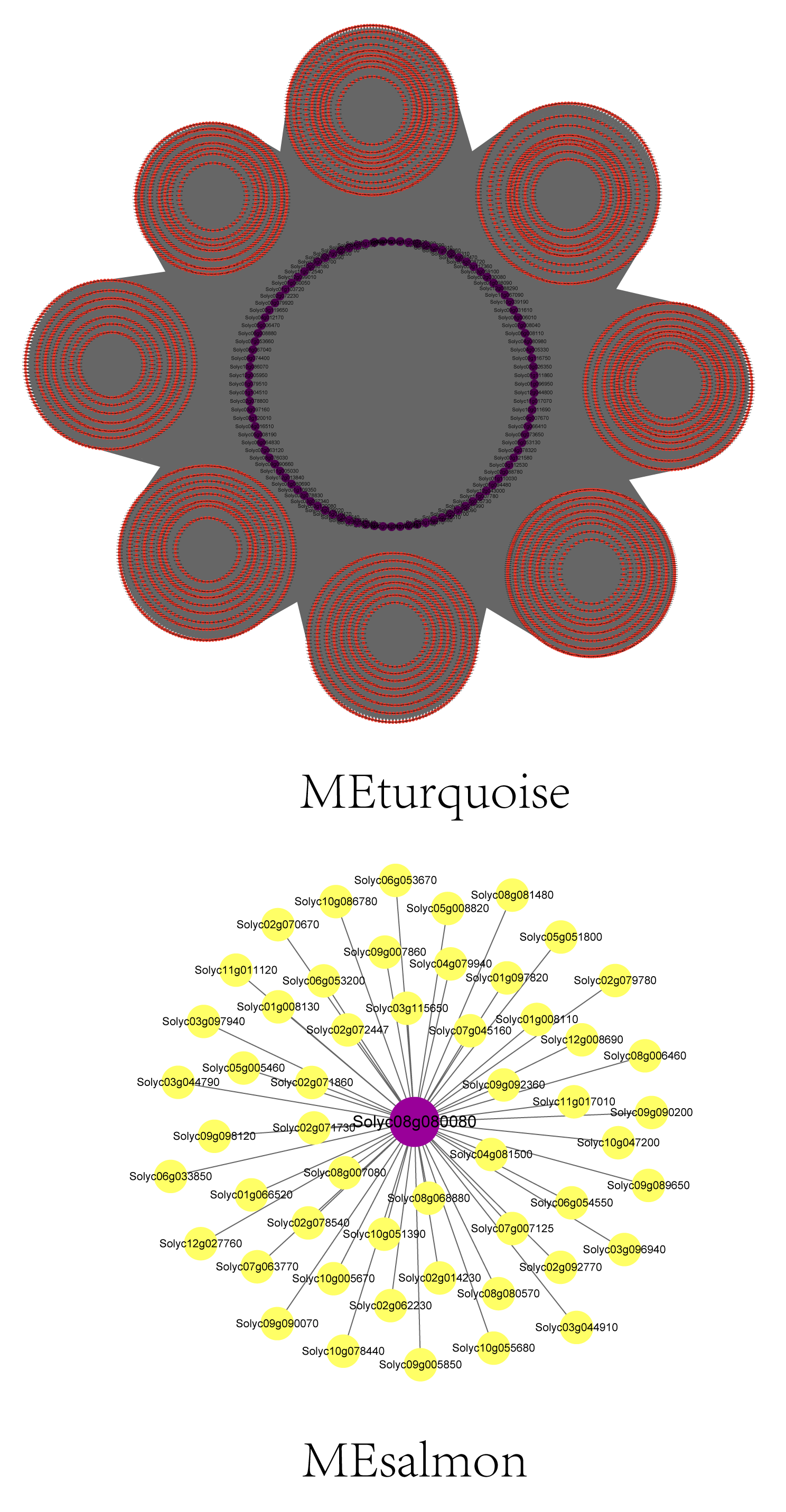

Supplement: Supplementary file 1 [file genes-14-01273-s001.zip › FigureS4. Genes co-expressed with tomato WD40 gene in MEturquoise module and MEsalmon module.tif]

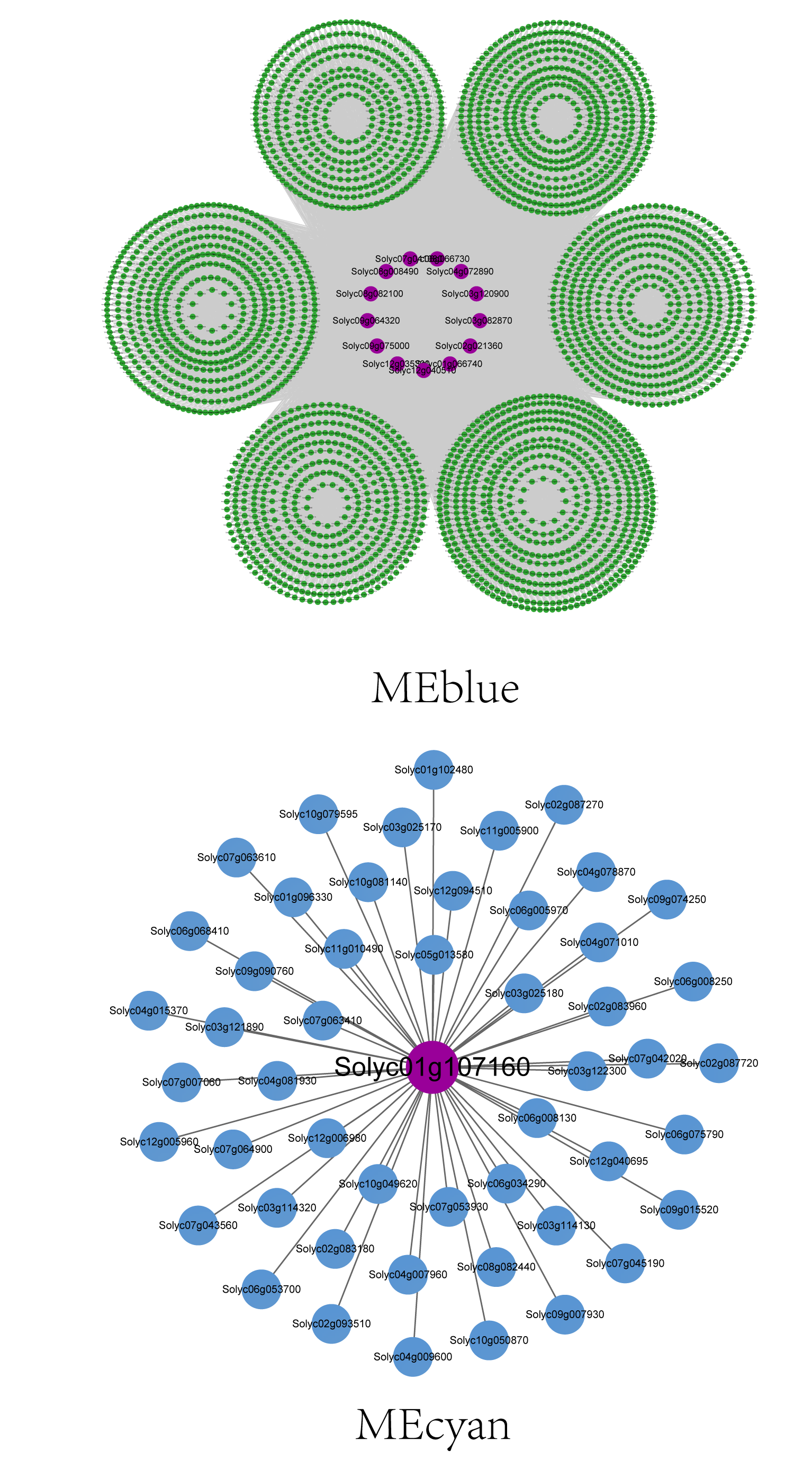

Supplement: Supplementary file 1 [file genes-14-01273-s001.zip › FigureS5. Genes co-expressed with tomato WD40 gene in MEblue module and MEcyan module.tif]
